# Differential diagnosis of lymphoma with ^18^F-FDG PET/CT in patients with fever of unknown origin accompanied by lymphadenopathy

**DOI:** 10.1007/s00432-023-04665-7

**Published:** 2023-03-08

**Authors:** Jia Chen, Dong Xu, Wen-Jin Sun, Wen-Xia Wang, Na-Na Xie, Qiu-Rong Ruan, Jian-Xin Song

**Affiliations:** 1 Department of Infectious Diseases, Tongji Hospital, Tongji Medical College, Huazhong University of Science and Technology, 1095 Jiefang Avenue, Wuhan, 430030 China; 2 Department of Infectious Diseases, Ezhou Central Hospital, Ezhou, 436099 China; 3 Department of Pediatric Hematology/Oncology, Sun Yat-Sen Memorial Hospital, Sun Yat-Sen University, Guangzhou, 528406 China; 4 Institute of Pathology, Tongji Hospital, Tongji Medical College, Huazhong University of Science and Technology, 1095 Jiefang Avenue, Wuhan, 430030 China

**Keywords:** Fever of unknown origin, Lymphoma, ^18^F-FDG PET/CT, Lymphadenopathy, Clinical parameters, Scoring system

## Abstract

**Purpose:**

To investigate the value of ^18^F-fluoro-2-deoxy-D-glucose positron emission tomography/computed tomography (^18^F-FDG PET/CT) in the differential diagnosis of lymphoma in patients with fever of unknown origin (FUO) accompanied by lymphadenopathy and to develop a simple scoring system to distinguish lymphoma from other etiologies.

**Methods:**

A prospective study was conducted on patients with classic FUO accompanied by lymphadenopathy. After standard diagnostic procedures, including PET/CT scan and lymph-node biopsy, 163 patients were enrolled and divided into lymphoma and benign groups according to the etiology. The diagnostic utility of PET/CT imaging was evaluated, and beneficial parameters that could improve diagnostic effectiveness were identified.

**Results:**

The sensitivity, specificity, positive predictive value (PPV), and negative predictive value (NPV) of PET/CT in diagnosing lymphoma in patients with FUO accompanied by lymphadenopathy were 81.0, 47.6, 59.3, and 72.7%, respectively. The lymphoma prediction model combining high SUVmax of the “hottest” lesion, high SUVmax of the retroperitoneal lymph nodes, old age, low platelet count, and low ESR had an area under the curve of 0.93 (0.89–0.97), a sensitivity of 84.8%, a specificity of 92.9%, a PPV of 91.8%, and an NPV of 86.7%. There was a lower probability of lymphoma for patients with a score < 4 points.

**Conclusions:**

PET/CT scans show moderate sensitivity and low specificity in diagnosing lymphoma in patients with FUO accompanied by lymphadenopathy. The scoring system based on PET/CT and clinical parameters performs well in differentiating lymphoma and benign causes and can be used as a reliable noninvasive tool.

**Registration number:**

This study on FUO was registered on http://www.clinicaltrials.gov on January 14, 2014, with registration number NCT02035670.

**Supplementary Information:**

The online version contains supplementary material available at 10.1007/s00432-023-04665-7.

## Introduction

Fever of unknown origin (FUO), originally defined by Petersdorf and Beeson in 1961, has more than 200 potential causes (Cunha et al. 2015; Haidar et al. 2022; Petersdorf et al. 1961). In China, various infections are still the main causes of FUO, followed by noninfectious inflammatory diseases (NIIDs) and neoplastic diseases (Wang et al. 2020). Almost all causes of FUO can be accompanied by lymphadenopathy. Lymphoma is one type of neoplasm that is classically associated with FUO, usually accompanied by lymphadenopathy, and has become one of the main causes of FUO in adults (Roca et al. 2007; Wu et al. 2022). The diagnosis of lymphoma remains challenging, because clinical manifestations and radiological features overlap with various infectious and inflammatory conditions. Timely diagnosis of lymphoma in patients with FUO is important for treatment and prognosis. However, assessments of lymph-node size and morphology are not reliable criteria, and a confirmed diagnosis of lymphoma mainly depends on histology (Fritscher-Ravens et al. 2000; Puri et al. 2003). Moreover, these invasive procedures are sometimes impossible to perform, and false-negative results are not uncommon in such procedures (Fiterman et al. 2022; Wang et al. 2022).

Promisingly, ^18^F-fluoro-2-deoxy-D-glucose positron emission tomography/computed tomography (^18^F-FDG PET/CT) shows great potential as a noninvasive diagnostic tool for FUO and is recommended for patients lacking potential diagnostic clues (PDCs) (Bleeker-Rovers et al. 2007; Gafter-Gvili et al. 2015; Georga et al. 2020; Hung et al. 2017; Kouijzer et al. 2018; Letertre et al. 2021; Sioka et al. 2015; Tokmak et al. 2014; Wang et al. 2019, 2020; Wright et al. 2021). Meta-analysis showed that the sensitivity of PET/CT in the diagnosis of FUO was between 86 and 98%, and the specificity was between 52 and 85% (Bharucha et al. 2017; Dong et al. 2011; Takeuchi et al. 2016, 2018). PET/CT has shown good performance regarding the management of patients with lymphoma (El-Galaly et al. 2018). However, despite its high sensitivity, the lack of desired specificity for malignancies makes it challenging to differentiate lymphomas with this approach in some cases (Makis et al. 2018; Nawwar et al. 2022).

This study aimed to analyze the clinical and PET/CT features of FUO patients with lymphadenopathy to identify factors that can predict lymphoma. Specifically, the purpose was to establish a combination of parameters best suited to distinguish lymphoma.

## Materials and methods

### Patients and standard diagnostic work-up

This prospective study recruited adult patients diagnosed with classic FUO accompanied by lymphadenopathy who were admitted to Tongji Hospital in Wuhan, Hubei Province, China, between January 2016 and August 2021. All enrolled patients underwent a predefined standard diagnostic work-up (Mulders-Manders et al. 2015).

Classic FUO was defined as follows: (1) duration of illness exceeding 3 weeks, (2) temperature exceeding 38.3 °C on more than three occasions, and (3) an inconclusive diagnosis despite appropriate investigation, with at least three outpatient visits or at least 3 days of hospitalization (Cunha et al. 2015). The exclusion criteria included nosocomial FUO, immunodeficiency-associated FUO, and travel-associated FUO. Pathological lymph nodes were defined as enlargement (greater than 10 mm on short-axis measurements) or hypermetabolism on PET/CT, dividing into superficial (cervical and supraclavicular, axillary, and inguinal) and central (mediastinal and retroperitoneal) lymph nodes according to site.

**Fig. 1:**
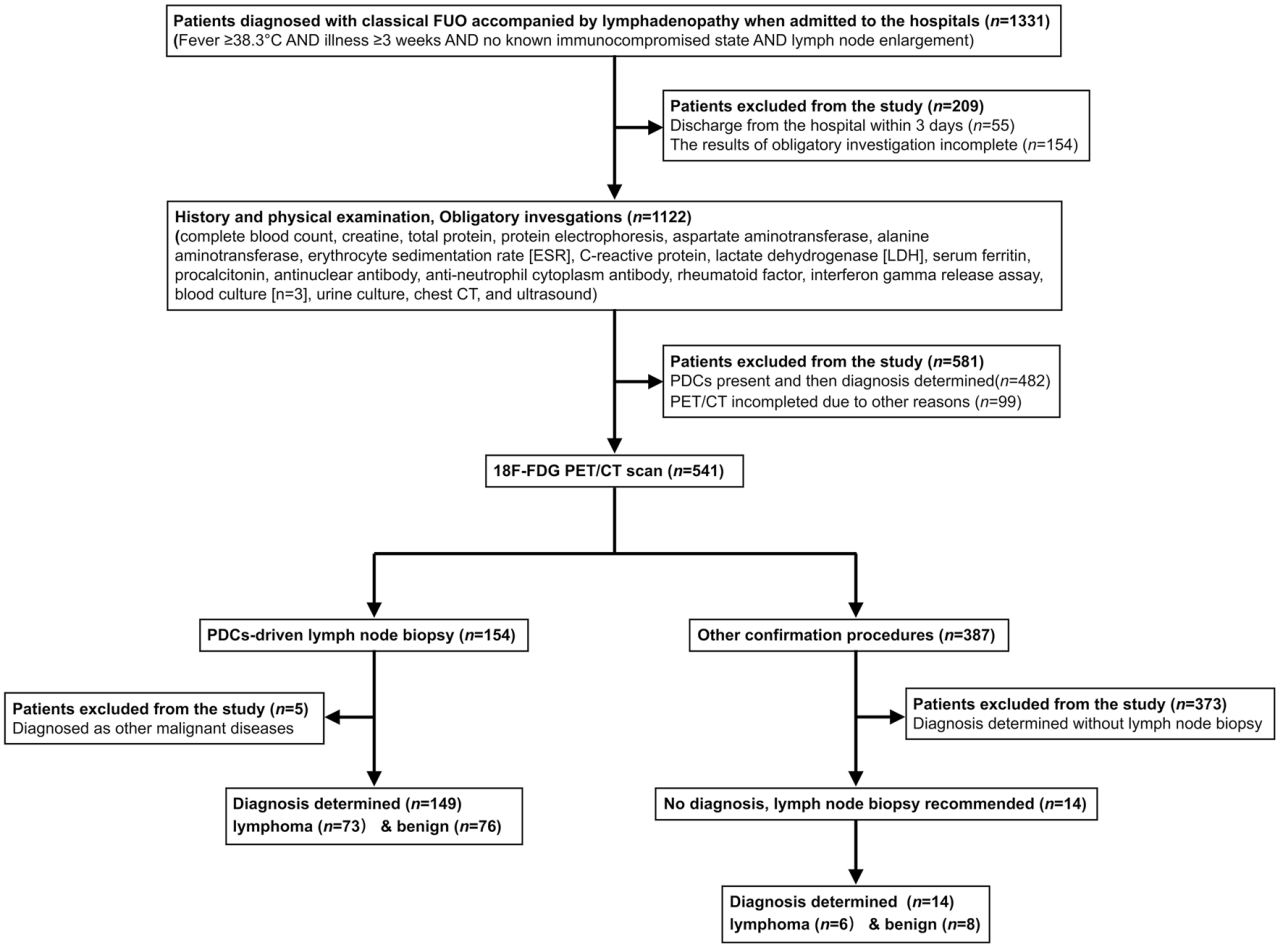
Flow diagram of participants of patients under the standard diagnostic work-up. Adult patients diagnosed as classic FUO accompanied by lymphadenopathy were recruited for predefined standard diagnostic work-up. Candidates who underwent PET/CT scan in the absence of PDCs and subsequently performed lymph-node biopsy as part of the confirmation procedure were eventually included

Standard diagnostic procedures included the following first steps: complete and repeated history taking, careful physical examination, and obligatory investigations (complete blood counts, aspartate aminotransferase, alanine aminotransferase, lactate dehydrogenase [LDH], C-reactive protein, creatine, total protein, protein electrophoresis, erythrocyte sedimentation rate [ESR], serum ferritin, procalcitonin, antinuclear antibodies, rheumatoid factor, urinalysis, interferon gamma release assay, blood culture [*n* = 3], urine culture, chest CT, and ultrasound) (Wang et al. 2020; Wu et al. 2018; Zhao et al. 2019). Complete blood counts and biochemical indexes were reexamined every 5 days during hospitalization to monitor the dynamic process. In the absence of PDCs, PET/CT examination was performed. Confirmation procedures (another imaging method, culture, biopsy or surgery) were then performed under PET/CT guidance. Patients who underwent lymph-node biopsy as part of the confirmation procedure were eventually enrolled (Fig. 1).

Based on the results of all standard procedures, the final diagnosis was determined by the research team in accordance with the relevant disease diagnostic guidelines. This was further confirmed by a 6-month follow-up. Five patients diagnosed with neoplastic diseases other than lymphoma were also excluded from the study. Finally, patients were divided into lymphoma and benign groups according to the etiology. The imaging characteristics of different etiologies and clinical significance of PET/CT were analyzed, and clinical parameters with potential predictive value were selected to construct a combined diagnostic model.

### ^18^F‐FDG PET/CT scan

PET/CT scans were performed on a PET/CT scanner (Discovery PET/CT 690, GE Healthcare) and followed standard technical requirements (i.e., fasting for at least 6 h, blood glucose levels below 11.1 mmol/l, and imaging performed 1 h after an injection of 2.96–4.44 MBq/kg of FDG). Emission and transmission images of the area between the mid-thigh and the base of the skull were acquired. Attenuation correction and image reconstruction were carried out with an iterative method. The maximum normalized uptake value (SUVmax) of the lesion was measured by the 3D region of interest technique to quantitatively evaluate FDG uptake. PET/CT images were independently evaluated by two experienced nuclear medicine physicians without access to the detailed clinical data. When abnormal FDG uptake is found on PET imaging, it is considered positive. Examination conclusions (including diagnostic opinions, diagnostic tendencies, or inconclusive results) were derived according to relevant guidelines. Any disagreements were resolved by consensus.

### Histopathologic examination

According to PET/CT results, hypermetabolic or suspicious lymph nodes were selected for biopsy. Peripheral lymph nodes were selected more often because of their availability, whereas central lymph nodes were seldom selected. If these procedures did not confirm the diagnosis, another tissue was selected for further biopsy. The tissue material was pathologically diagnosed independently by two experienced pathologists and grouped according to the World Health Organization (WHO) Classification of Lymphoma (Swerdlow et al. 2016). Any disagreements were resolved by consensus.

### Statistical analysis

All statistical analyses were performed using SPSS Statistics 26.0 Software (IBM, Chicago). Categorical variables are expressed as frequencies and percentages (*n*%), and continuous variables are shown as medians and interquartile ranges [IQRs]. According to the theoretical frequency, the *χ*2 or Fisher’s exact test was used to compare the classification variables between the groups. According to the data distribution, Student’s *t* test or the Mann‒Whitney *U* test was used to compare the intergroup continuous variables. All statistical tests were two-sided with a significance level of *p* < 0.05.

Considering lymphoma as the outcome variable, univariate analysis was used to screen potential diagnostic indicators. All candidate variables were included in multivariate analysis to determine the indicators eligible for inclusion in the model. Multivariate analysis results are presented as odds ratios (ORs) and 95% confidence intervals (CIs). ﻿﻿The area under the receiver-operating characteristic (ROC) curve (AUC) was used to evaluate the performance of the prediction model.

## Results

### Patient characteristics

Seventy-nine patients were eventually diagnosed with lymphoma (mainly non-Hodgkin lymphoma); the common pathological types were diffuse large B-cell lymphoma (25 cases), angioimmunoblastic T-cell lymphoma (12 cases), extranodal NK/T-cell lymphoma, nasal type (12 cases), and peripheral T-cell lymphoma, not otherwise specified (9 cases). Eighty-four patients were diagnosed with nonneoplastic disease, including 30 cases of infectious diseases, 24 cases of NIID, and 19 cases of other categories. The common benign causes included histiocytic necrotizing lymphadenitis (17 cases), adult-onset Still's disease (AOSD) (13 cases), and infection with *tubercle bacillu*s (7 cases) or Epstein‒Barr virus (7 cases) (detailed etiologies are listed in supplementary table 1).

The clinical parameters of patients with different etiologies were compared, including general characteristics, complete blood counts, biochemical indexes, and routine imaging findings (Table 1). Those in the lymphoma group were more likely to be male and older. In the lymphoma group, thrombocytopenia, decreased albumin, and elevated LDH and carcinoembryonic antigen were observed. ESR was elevated in the benign group. Hepatomegaly and splenomegaly were more common in malignant patients. In this study, the proportion of superficial lymph-node enlargement was higher in the benign group. The sizes of superficial and central lymph nodes in the lymphoma group were larger than those in the benign group. Seventeen patients showed signs of lymph-node fusion, but there was no significant difference between the two groups.

**Table 1:** Clinical parameters of patients with different etiologies

	Lymphoma group (*n* = 79)	Benign group (*n* = 84)	*P* value
Sex male	51 (64.6%)	39 (46.4%)	0.020
Age (years)	57 [44–65]	31 [21–45]	0.000
Duration of fever (days)	39 [28–52]	33 [25–48]	0.118
Weight loss	29 (36.7%)	20 (23.8%)	0.073
White blood cell count (*10^9^/L)	4.9 [3.3–8.1]	6.7 [3.4–10.5]	0.069
Neutrophil count (*10^9^/L)	3.4 [1.9–6.1]	4.6 [2.2–8.5]	0.114
Hemoglobin (g/L)	102 [89–118]	109 [95–122]	0.077
Platelet count (*10^9^/L)	142 [83–206]	202 [146–282]	0.000
Albumin (g/L)	31.0 [26.9–34.7]	35.1 [31.1–39.8]	0.000
LDH (U/L)	414 [290–662]	32 l [206–548]	0.008
ESR (mm/H)	26 [9–66]	45 [26–81]	0.006
Carcinoembryonic antigen (ng/mL)	2.1 [1.4–3.1]	1.4 [1.0–2.1]	0.000
Hepatomegaly	16 (20.3%)	5 (6.0%)	0.006
Spleen thickness (cm)	4.7 [3.7–5.1]	4.2 [3.6–4.5]	0.002
Superficial LN enlargement	53 (67.1%)	77 (91.7%)	0.000
Superficial LN size (cm)	1.6 [0.9–2.4]	1.1 [0.8–1.5]	0.010
Central LN enlargement	55 (69.6%)	59 (70.2%)	0.931
Central LN size (cm)	2.2 [1.2–3.0]	1.5 [1.2–1.8]	0.052
Fusion of lymph nodes	10 (12.7%)	7 (8.3%)	0.367

*N* (%), median [IQR]

*LDH* Lactate dehydrogenase, *ESR* Erythrocyte sedimentation rate, *LN* Lymph node

### PET/CT characteristics and performance in diagnosing FUO

**Fig. 2:**
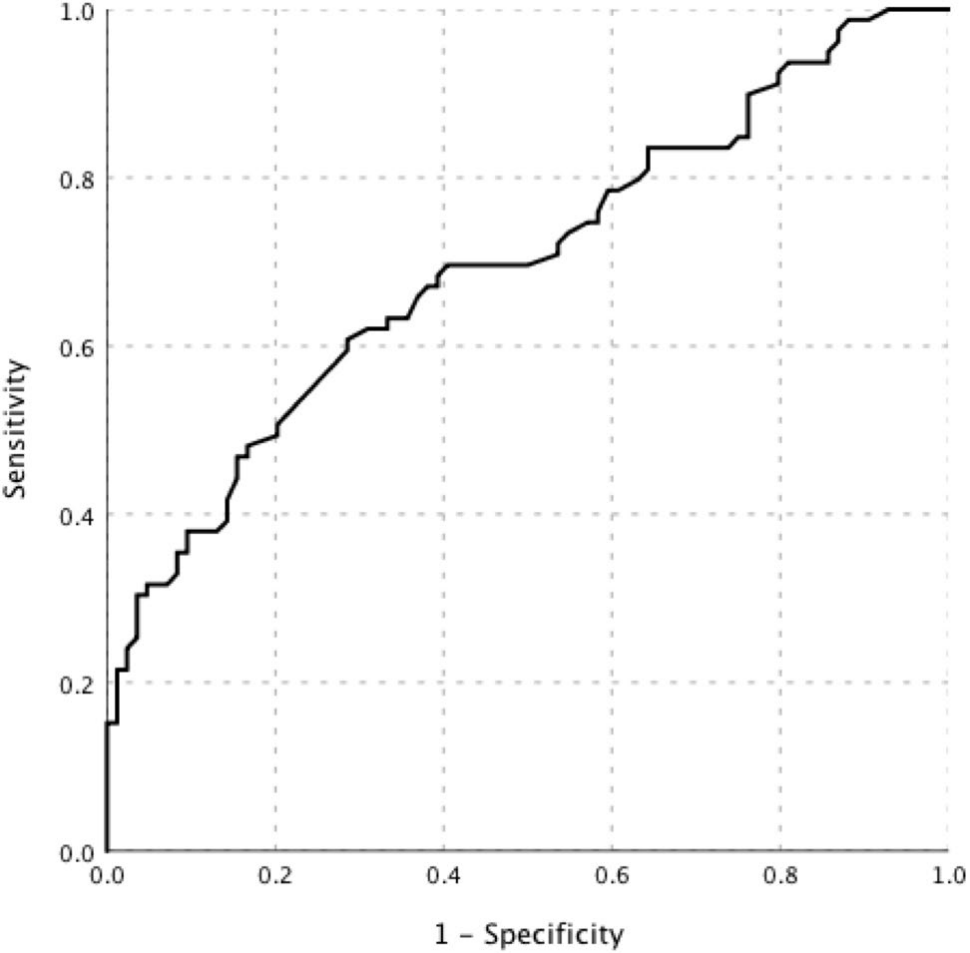
The ROC curve of SUVmax value in diagnosing lymphoma in patients with FUO accompanied by lymphadenopathy. The AUC of SUVmax was 0.70 (95% CI, 0.62–0.78)

The PET/CT examinations showed positive findings in all patients. Lymph nodes (69.9%, 114/163) were the most common site with the highest uptake of FDG throughout the body (the “hottest” lesions). The intensity of FDG uptake in patients with lymphoma was significantly higher than that in patients with other diseases (Table 2). The AUC of SUVmax in diagnosing lymphoma was 0.70 (95% CI, 0.62–0.78) (Fig. 2).

**Table 2:** PET/CT characteristics of patients with different etiologies

	Lymphoma group (*n* = 79)	Benign group (*n* = 84)	*P* value
SUVmax (the “hottest” lesion)	12.0 [6.7–17.6]	7.3 [5.3–10.1]	0.000
SUVmax of liver ratio	4.1 [2.3–6.0]	2.5 [1.8–3.5]	0.000
LN hypermetabolic	76 (96.2%)	84 (100%)	0.149
LN SUVmax	9.7 [4.3–15.0]	7.2 [4.7–9.7]	0.030
Superficial LN hypermetabolic	67 (84.8%)	83 (98.8%)	0.001
Superficial LN SUVmax	8.7 [4.1–13.4]	6.5 [4.1–9.3]	0.156
Central LN hypermetabolic	65 (82.3%)	64 (76.2%)	0.339
Central LN SUVmax	9.5 [4.2–15.8]	6.7 [4.8–8.7]	0.014
Retroperitoneal LN SUVmax	10.1 [4.3–16.9]	6.4 [3.9–8.8]	0.001
Spleen hypermetabolic	46 (58.2%)	42 (50.0%)	0.292
Spleen SUVmax	6.3 [3.9–8.8]	3.8 [3.0–4.9]	0.000
Bone marrow hypermetabolic	37 (46.8%)	23 (27.4%)	0.010
Bone marrow SUVmax	6.3 [4.1–9.8]	4.5 [3.7–5.2]	0.008

*N* (%), median [IQR]

*PET/CT* Positron emission tomography/computed tomography, *SUVmax* The maximum standardized uptake value, *LN* Lymph node

In this study, 98.2% (160/163) of patients showed FDG avidity in the lymph nodes. The benign group had a higher proportion of increased superficial lymph-node metabolism. Although the proportion of elevated metabolism in central lymph nodes was similar between the two groups, the corresponding SUVmax was higher in the lymphoma group. Especially, in the retroperitoneal lymph nodes, the uptake value of the lymphoma group was significantly higher than that of the benign group. The metabolic characteristics of two common benign diseases (histiocytic necrotizing lymphadenitis and AOSD) were compared with lymphoma in detail (Table 3). The SUVmax values of the “hottest” lesions in lymphoma group were significantly higher than that in AOSD group. There was no significant difference between the three groups in the proportion of elevated lymph-node metabolism and the corresponding SUVmax value.

**Table 3:** The metabolic characteristics of lymphoma, histiocytic necrotizing lymphadenitis, and AOSD

	Lymphoma (*n* = 79)	Histiocytic necrotizing lymphadenitis (*n* = 17)	AOSD (*n* = 13)	*P* value
SUVmax (the “hottest” lesion)	12.0 [6.7–17.6] ^a^	9.7 [7.4–13.5] ^a,b^	6.4 [4.9–9.9] ^b^	0.018
LN hypermetabolic	76 (96.2%)	17 (100%)	13 (100%)	0.557
LN SUVmax	9.7 [4.3–15.0]	9.7 [7.4–13.5]	6.4 [4.6–9.9]	0.432
Superficial LN enlarged	53 (67.1%) ^a^	17 (100%) ^b^	11 (84.6%) ^a,b^	0.013
Superficial LN size	1.6 [0.9–2.4]	1.2[1.0–1.7]	1.0 [0.6–1.3]	0.092
Superficial LN hypermetabolic	67 (84.8%)	16 (94.1%)	13 (100%)	0.207
Superficial LN SUVmax	8.7 [4.1–13.4]	8.8 [5.9–13.8]	5.9 [4.4–7.9]	0.348
Central LN enlarged	55 (69.6%)	13 (76.5%)	7 (53.8%)	0.397
Central LN size	1.9 [1.3–3.0]	1.3[1.2–1.6]	1.3[1.2–1.5]	0.121
Central hypermetabolic	65 (82.3%)	13 (76.5%)	10 (76.9%)	0.802
Central LN SUVmax	9.5 [4.2–15.8]	8.7 [6.3–9.9]	6.2 [3.8–7.7]	0.213

*N* (%), median [IQR]

*AOSD* Adult-onset still’s disease, *SUVmax* The maximum standardized uptake value, *LN* Lymph node

^a,b^Pairwise comparison between subgroups, *P* values < 0.05

The lymphoma group had a higher proportion of increased bone marrow metabolism and a higher corresponding SUVmax value. The proportion of splenic uptake was similar in the two groups, but the spleen SUVmax value was higher in the lymphoma group.

Markedly FDG-avid lymphadenopathy and diffuse marrow and splenic uptake were demonstrated on PET/CT, most suggestive of lymphoma. In the malignancy group, 64 patients were considered to have lymphoma by PET/CT, and 15 patients were not considered to have lymphoma or their status could not be determined. In the benign group, 40 patients were considered to have nonmalignant disease by PET/CT, and 44 patients could not be excluded for lymphoma (including 11 cases of histiocytic necrotizing lymphadenitis and 6 cases of AOSD). The sensitivity, specificity, positive predictive value (PPV), and negative predictive value (NPV) of PET/CT in the diagnosis of lymphoma in patients with FUO accompanied by lymphadenopathy were 81.0, 47.6, 59.3, and 72.7%, respectively.

### Histological results

The final puncture sites included cervical and supraclavicular (116 cases), axillary (19 cases), inguinal (13 cases), mediastinal (5 cases), and retroperitoneal (10 cases) lymph nodes. For the final selected lymph nodes, there was no difference in size or uptake value between the benign and lymphoma groups (*p* < 0.05). The results of lymph-node biopsy were of diagnostic significance in 92 patients, including 61 patients with lymphoma, 17 patients with histiocytic necrotizing lymphadenitis, 6 patients with Epstein–Barr virus infection, 4 patients with *tubercle bacillu*s infection, and 4 patients with noninfectious inflammatory disease. The remaining patients showed nonspecific reactive hyperplasia or no obvious disease manifestations.

Ultimately, 149 patients underwent bone marrow smear or biopsy. Sixteen cases of lymphoma were diagnosed by bone marrow examination. Another 11 patients showed abnormal lymphocytosis in the bone marrow and were eventually diagnosed with lymphoma. Other patients manifested nonspecific findings, such as abnormal bone marrow proliferation, hemophagocytosis, indeterminate abnormal cells, or no significant findings. Fourteen patients underwent biopsy of the nasopharynx, kidney, adrenal gland, liver, lung, or other tissues, of which 13 were diagnosed with lymphoma and 1 was diagnosed with tuberculosis.

### Mathematical model construction

PET/CT features and clinical parameters were included in the screening to select beneficial predictors that would improve diagnostic effectiveness. Relevant blood indicators included values measured at admission and limits over the course of disease. To facilitate clinical use, continuous variables were converted to categorical variables.

**Fig. 3:**
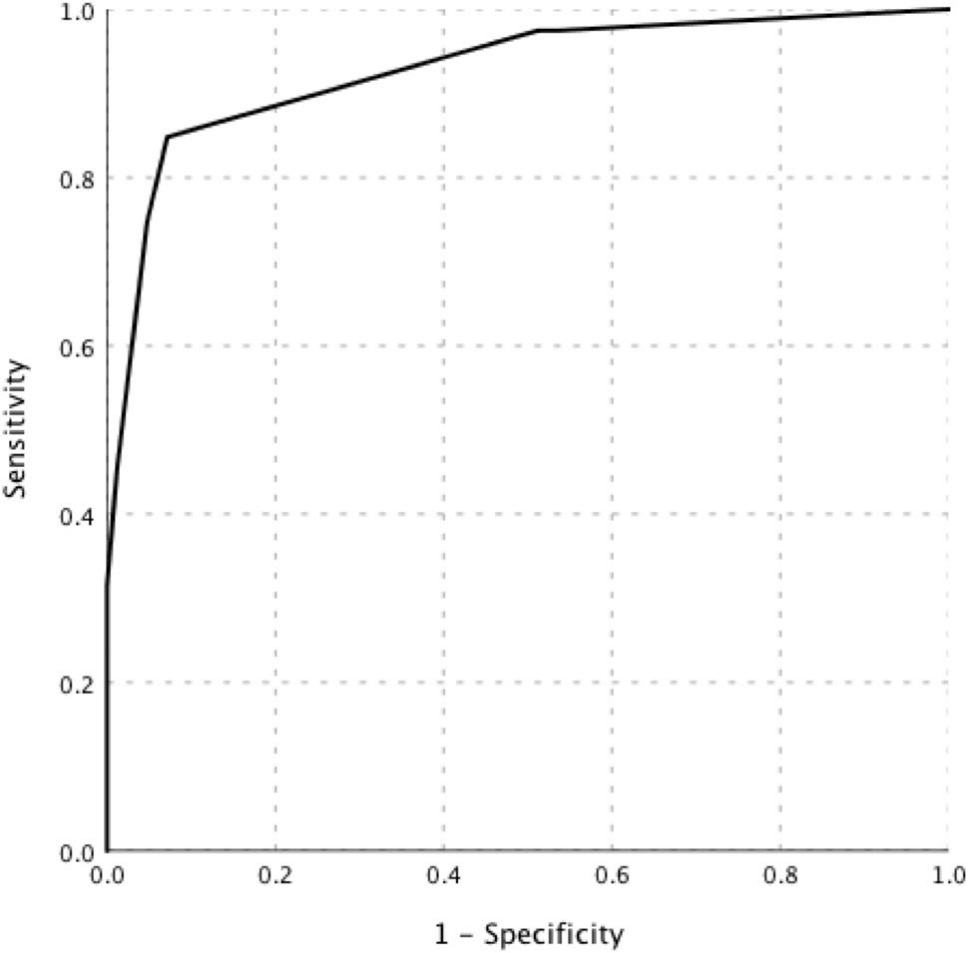
The ROC curve of the scoring system in diagnosing lymphoma in patients with FUO accompanied by lymphadenopathy. The AUC of the scoring system was 0.93 (95% CI, 0.89–0.97)

Binary logistic regression analysis showed that the following five indicators contributed to the diagnosis of lymphoma: high SUVmax of the “hottest” lesion (SUVmax of liver ratio ≥ 5), high SUVmax of retroperitoneal lymph nodes (retroperitoneal lymph-node SUVmax ≥ 9.4), old age (age ≥ 53 years), low platelet count (maximum platelet count < 200*10^9^/L), and low ESR (maximum ESR ≤ 22 mm/H) (Table 4). By rounding the coefficients of each variable in the model, the score was calculated, as presented in Table 4. The best cut-off point was 4; the AUC of the scoring system was 0.93 (95% CI, 0.89–0.97), and the sensitivity, specificity, PPV, and NPV were 84.8, 92.9, 91.8, and 86.7%, respectively (Fig. 3). The higher the score is, the greater the probability of lymphoma. In a simple assessment, patients were considered less likely to have lymphoma if they met only one or none of the indicators.

**Table 4:** Multiple regression analysis for diagnosis of malignancy in FUO patients

Variable	β-Coefficient	Standard error	*P* value	OR (95% CI)	Score
High SUVmax of liver ratio	2.01	0.73	0.006	7.49 (1.78–31.58)	2
High SUVmax of the retroperitoneal LN	2.51	0.71	0.000	12.33 (3.06–49.63)	3
Old age	2.85	0.61	0.000	17.29 (5.21–57.45)	3
Low ESR	2.70	0.65	0.000	14.92 (4.15–53.67)	3
Low platelet count	2.66	0.59	0.000	14.27 (4.53–44.91)	3

*OR* Odds ratio, *CI* Confidence interval, *SUVmax* The maximum standardized uptake value, *LN* Lymph node, *ESR* Erythrocyte sedimentation rate

## Discussion

Due to the complexity and diversity of FUO-related diseases, identifying the etiologies remains a challenge for contemporary clinicians. Patients with FUO often have lymphadenopathy, which may be caused by various factors, such as lymphoma, lymph nodal tuberculosis, histiocytic necrotizing lymphadenitis, or AOSD. The final etiological classification in this study is consistent with that in other studies, and lymphoma was the most common malignancy (Wright et al. 2022). In the benign group, the highest proportion were infectious diseases, including a variety of different pathogens such as *tubercle bacillu*s and Epstein–Barr virus. Although the lymphoma group presented with thrombocytopenia, decreased albumin, elevated lactate dehydrogenase and carcinoembryonic antigen, hepatomegaly, and splenomegaly, these features did not make a diagnosis separately. In addition, lymph-node features in the lymphoma and benign groups were not sufficient to aid diagnosis.

The high sensitivity and wide visual field of PET/CT facilitates the detection of clinically unknown or undetectable lesions with the conventional imaging techniques and the acquisition of multisystem observations (Besson et al. 2016; Kouijzer et al. 2018). Compared with the pathological results, PET/CT correctly diagnosed 104 cases, with a sensitivity of 81.0% and a specificity of 47.6%. SUV showed high sensitivity but low specificity and diagnostic yield in differentiating benign and malignant disease (Rayamajhi et al. 2016; Wang et al. 2018). Consistent with the results of previous studies, it was not possible to judge benign and malignant lymph nodes only according to the SUVmax value.

In patients with FUO, the difference in lymph-node metabolic characteristics between the benign and lymphoma groups was only reflected in the higher SUV value of central lymph nodes in the lymphoma group. Especially, for superficial lymph nodes, patients with benign causes, such as histiocytic necrotizing lymphadenitis and AOSD, can also present with hypermetabolism (Erhamamci et al. 2014; Zhou et al. 2020). This study found that the lymph-node metabolic characteristics of these two types of benign patients were similar to those of lymphoma, and they were often misdiagnosed as lymphoma by PET/CT. It is difficult to distinguish benign from malignant lymphadenopathy on imaging.

Although the lymph nodes with the highest metabolic value that were easily achievable were selected for puncture under the guidance of PET/CT, false-negative results were obtained in 18 patients, and meaningless results were obtained in 53 patients. Only 56.4% (92/163) of patients in this study benefited from lymph-node biopsy. Among 149 patients with bone marrow examination, only 27 received diagnostic information. Consistent with the previous studies, the effectiveness of these invasive histological examinations in differentiating benign from malignant lesions is not satisfactory, and their diagnostic value for nonneoplastic diseases is limited (Letertre et al. 2021; Wang et al. 2019; Hong et al. 2019). Many patients undergo invasive surgery with only nonspecific reactive hyperplasia or no detectable findings.

Wang et al. demonstrated that combined clinical parameters improve the diagnostic efficacy of PET/CT in patients with FUO (Wang et al. 2020). However, there are few studies on FUO accompanied by lymphadenopathy, and no consensus has been reached on how to combine the parameters in this population. Different from previous studies that only focused on blood indicators at admission, this study monitored the dynamic changes of these indicators and found that the limit values were more conducive to differential diagnosis, further confirming the importance of dynamic observation in the diagnosis of FUO. The indexes selected to construct the prediction model were consistent with the clinical characteristics of lymphoma. It is well known that the elderly has a high incidence of malignancy. Patients with lymphoma often present with a significant increase in FDG uptake and a high incidence of retroperitoneal lymph-node involvement. Previous studies have shown that lymphoma patients present with a high incidence of thrombocytopenia and a low incidence of elevated ESR concentration (Wu et al. 2022). ESR reflects the existence and extent of infection and inflammation, which is commonly elevated in patients with infectious disease and NIID (Okuyucu et al. 2018; Wan et al. 2023). Through this prospective study in China, favorable factors for the etiological classification of FUO accompanied by lymphadenopathy were screened out, and the weight of each parameter was further analyzed to compose the formula. The combination of high SUVmax and clinical parameters can help distinguish lymphoma from other etiologies and optimize diagnostic strategies. This approach had a sensitivity of 84.8%, a specificity of 92.9%, and an AUC of 0.93. When the PET/CT scan fails to diagnose the cause of FUO, the scoring system can be used to calculate the lymphoma probability, rapidly and accurately distinguish the benign and malignant etiologies of FUO, optimize the diagnosis strategy, and avoid unnecessary invasive procedures.

The strengths of our study include a prospective setting, the combination of clinical parameters and PET/CT imaging to further improve diagnostic efficacy, and the establishment of a simple scoring system. The lymphoma prediction model was established according to the characteristics of PET/CT, which further improved its diagnostic efficiency in FUO. The scoring system performed satisfactorily in differentiating malignant and benign lesions. When patients have atypical clinical features and lack diagnostic clues, the lymphoma prediction model can help clinicians improve the accuracy of FUO diagnosis, especially to avoid unnecessary invasive procedures.

Our study cohort was not sufficiently large. The single-center design likely limits the robustness of our findings. In particular, the small number of cases of histiocytic necrotizing lymphadenitis and AOSD may affect the results of the comparison with lymphoma characteristics. Multicenter and larger-sample studies are needed to further verify the value of our prediction model for routine use in clinical practice.

## Conclusions

^18^F-FDG PET/CT has moderate sensitivity and low specificity in the diagnosis of FUO accompanied by lymphadenopathy, and its diagnostic efficiency can be improved by combining clinical parameters. The scoring system based on PET/CT scans and clinical parameters shows excellent performance in differentiating benign and malignant causes of FUO, and can be used as a simple and reliable tool. Especially, in patients who lack PDCs or require invasive procedures, this formula can be used to calculate the likelihood of lymphoma and make a diagnosis rapidly and accurately.

## Supplementary Information

Below is the link to the electronic supplementary material.

Supplementary Information Table S1 (DOCX 18 KB)

## Data Availability

The data that support the findings of this study are available from the corresponding author upon reasonable request.
