# Supplementary material for: Differential diagnosis of lymphoma with 18F-FDG PET/CT in patients with fever of unknown origin accompanied by lymphadenopathy
Source: J Cancer Res Clin Oncol. 2023 Mar 8;149(10):7187–96. doi: 10.1007/s00432-023-04665-7 (PMC10374793; doi:10.1007/s00432-023-04665-7)
Supplement: Supplementary file 1 — Supplementary Information Table S1 (DOCX 18 KB) [file 432_2023_4665_MOESM1_ESM.docx]

**Journal:** [**Journal of Cancer Research and Clinical Oncology**](https://www.springer.com/journal/432/?IFA)

**Differential diagnosis of lymphoma with ^18^F-FDG PET/CT in patients with fever of unknown origin accompanied by lymphadenopathy**

Jia Chen; Dong Xu; Wen-Jin Sun; Wen-Xia Wang; Na-Na Xie; Qiu-Rong Ruan^*^; Jian-Xin Song^*^

***Corresponding author**: **Jian-Xin Song**, [songsingsjx@sina.com](mailto:songsingsjx@sina.com), Department of Infectious Diseases, Tongji Hospital, Tongji Medical College, Huazhong University of Science and Technology, Wuhan 430030, China.

**Qiu-Rong Ruan**, [ruanqiurong@sina.com](mailto:ruanqiurong@sina.com), Institute of Pathology, Tongji Hospital, Tongji Medical College, Huazhong University of Science and Technology, Wuhan 430030, China

**Table S1. Clinical diagnosis and etiological classification in 163 patients with FUO.**

| Etiology classification | Case number | Clinical diagnosis |
| --- | --- | --- |
| Lymphoma | 79 | Diffuse large B-cell lymphoma (25), angioimmunoblastic T-cell lymphoma (12), extranodal NK/T-cell lymphoma, nasal type (12), peripheral T-cell lymphoma, not otherwise specified (9), anaplastic large cell lymphoma (5), classic Hodgkin lymphoma (2), γδT cell lymphoma (1), aggressive NK cell leukemia/lymphoma (1), un-classification (12) |
| Infection | 30 | *Tubercle bacillus* (7), *Epstein–Barr virus* (7), *Candida albicans* (2), *Mycoplasma pneumoniae* (2), *Staphylococcus* (3), *Micrococcus luteus* (1), *chlamydia* (1), uncertain (7) |
| NIID | 24 | AOSD (13), undifferentiated connective tissue disease (3), vasculitis (1), primary hemophagocytic syndromes (1), systemic lupus erythematosus (1), Sjogren's syndrome (1), EVANS syndrome (1), macrophage activation syndrome (1), IgG-related disease (1), mixed connective tissue disease (1) |
| Miscellaneous | 19 | Histiocytic necrotizing lymphadenitis (17), subacute thyroiditis (1), drug allergy (1) |
| No diagnosis | 11 | uncertain |

FUO, fever of unknown origin; NIID, noninfectious inflammatory disease; AOSD, adult-onset still’s disease.
